# Modern Sociology

**Published:** 1899-04-15

**Authors:** 


### Modern Sociology.

### THE MARRIAGE OF THE UNFIT.

This is one of the subjects which keeps continually recurring in both medical and sociological literature, from the days of Lycurgus, through Sir Thomas More's "Utopia," down to our own times. The unanimity of opinion upon it among experts is most striking. All refer to its prevention regretfully as a "counsel of perfection," unattainable only on account of human ignorance and prejudice. The desirability of preventing the marriage of the unfit by legal means if possible is taken for granted, and the only thing against such measures is the immense practical difficulty of their enforcement. But somehow little or nothing has been done about it since the Spartan experiment—which collapsed. Without attempting to decide whether this inactivity was masterly or otherwise we would like to briefly call attention to a few considerations which we think are hardly given sufficient weight in the discussion of this problem. First of all is the extreme difficulty of defining the term "unfit." There are at least three kinds of unfitness—physical, mental, and moral—and unfortunately, or fortunately, they seldom all occur in one individual. The prize-fighter and the Hooligan may be extremely fit physically, which may be all the fitness required of them by society, but mentally and morally the less said of them the better. On the other hand, the philosopher, the Rousseau, a model of fitness mentally, may be decidedly lacking in both physique and morals. Yet, are not both the prize-fighting navvy and the thin, sour philosopher useful members of society, in spite of their grave deficiencies? Should we not risk much and gain very little by forbidding either of them to marry? And yet, a man who was confessedly unfit in two out of three of his essential relations would certainly have to be ruled out by any formal standard which could be adopted for application. There was a world of truth in Huxley's shrewd, if cynical observation, that fitness is merely ability to survive, and we call those "fittest" which *have* survived in any given case. And who can tell what will be the next change of environment to which the human race will have to respond, and which of us will be fittest to promptly adjust himself to it? There is the soundest biological basis for the "pawky" dictum of Aunt Dorcas, "It takes all sorts o' people to make up a world," even of such as will prompt in us her pious pendant, "and I thanks the Lord I ain't one of 'em."

Supposing we were to take a perfectly simple standard and forbid the marriage of the habitual criminal. Three serious considerations would present themselves at once. First, that the criminal classes either marry (or more commonly form voluntary connections, to which both parties are often curiously faithful) very young, and in many cases would already have offspring before they have been convicted a sufficient number of times to be classed as "habitual." Second, that the fertility of these people is already low, and the death-rate among their children extremely high, from disease, exposure, and neglect. Third, that the "habitual criminal" is an anachronism and an absurdity which ought to be no longer tolerated in a civilised community. Upon the third conviction he should be promptly committed to a reformatory, under indeterminate sentence, there to remain until he either reforms or dies. Mr. Brockway's results at Elmira (New York) have shown that 80 per cent. of even "criminals born" reform within four years when once they understand that they are committed until they do, and seldom need a second "course." So that rational treatment of the criminal would practically eliminate this danger without the need of such drastic measures. Behind all which, of course, lies the brutal fact that to forbid the criminal to marry is simply to quadruple his illicit connections. He would be only too glad of the excuse.

Now let us look at the question of prohibition upon physical grounds. It would, of course, be absurd to adopt a standard of height, weight, or muscular strength, or any tests of physical development. Nor would our darling conception, "a good constitution," be of any value, for it would take a genius to either define it or to detect its absence with certainty. Many a puny child and pale stripling will outwork and outlive his sturdier-looking brother, and after being "delicate" for half a lifetime, will grow into a vigorous middle age and die over seventy. As for "disease tendencies," nearly all that are definable are really early stages of the disease itself; so that practically nothing but the presence of actual disease could be relied upon as a bar, and this bar is to a considerable extent already operative. The only exceptions which we think could reasonably be raised to this rule would be the cases in which there was a family history of consumption, of epilepsy, or of insanity. Syphilis, of course, enforces its own prohibitions, and permits comparatively few infected offspring to survive. As to consumption, its heredity has been pretty thoroughly disproved, the only thing transmitted being a certain degree of predisposition to the attack of the bacillus. How great this is is quite unsettled; but the few figures obtainable from insurance reports indicate that it seldom exceeds 15 or 20 per cent. And are we justified in forbidding the banns of an intelligent, honourable, and industrious young man or woman simply on the grounds that the chances are one in five in favour of his developing the disease, and that in that case one-fifth of his offspring may possibly develop it? A brief mathematical process will show the chances of the disease appearing in his children to be one in twenty-five. And of "consumptive" families have come some of our choicest spirits—Keats, Shelley, Hood, Robert Louis Stevenson.

As for epilepsy, if the disease has not developed before marriageable age the chances of its appearing afterwards are very small, no matter how unfavourable the family history, and while it has probably greater "carrying powers" than almost any other disease, yet the reasonable probabilities of its appearance in another generation, though they might lead us to discourage marriage, are hardly great enough to justify us in forbidding it by law.

